# SIGMA^2^: A system for the integrative genomic multi-dimensional analysis of cancer genomes, epigenomes, and transcriptomes

**DOI:** 10.1186/1471-2105-9-422

**Published:** 2008-10-07

**Authors:** Raj Chari, Bradley P Coe, Craig Wedseltoft, Marie Benetti, Ian M Wilson, Emily A Vucic, Calum MacAulay, Raymond T Ng, Wan L Lam

**Affiliations:** 1 Department of Cancer Genetics and Developmental Biology, BC Cancer Agency Research Centre, Vancouver, BC, Canada; 2 Department of Cancer Imaging, BC Cancer Agency Research Centre, Vancouver, BC, Canada; 3 Department of Computer Science, University of British Columbia, Vancouver, BC, Canada

## Abstract

**Background:**

High throughput microarray technologies have afforded the investigation of genomes, epigenomes, and transcriptomes at unprecedented resolution. However, software packages to handle, analyze, and visualize data from these multiple 'omics disciplines have not been adequately developed.

**Results:**

Here, we present *SIGMA*^2^, a system for the integrative genomic multi-dimensional analysis of cancer genomes, epigenomes, and transcriptomes. Multi-dimensional datasets can be simultaneously visualized and analyzed with respect to each dimension, allowing combinatorial integration of the different assays belonging to the different 'omics.

**Conclusion:**

The identification of genes altered at multiple levels such as copy number, loss of heterozygosity (LOH), DNA methylation and the detection of consequential changes in gene expression can be concertedly performed, establishing *SIGMA*^2 ^as a novel tool to facilitate the high throughput systems biology analysis of cancer.

## Background

Multiple mechanisms of gene disruption have been shown to be important in the development of cancer. Genetic alterations (mutations, changes in gene dosage, allele imbalance) and epigenetic alterations (changes in DNA methylation and histone modification states) are responsible for changing the expression of genes. High throughput approaches have afforded the ability to interrogate the genomic, epigenomic and gene expression (transcriptomic) profiles at unprecedented resolution [1-6]. However, a gene can be disrupted by one or by a combination of mechanisms, therefore, investigation in a single 'omics dimension (genomics, epigenomics, or transcriptomics) alone cannot detect all disrupted genes in a given tumor. Moreover, individual tumors may have different patterns of gene disruption, by different mechanisms for a given gene while achieving the same net effect on phenotype. Hence, a multi-dimensional approach is required to identify the causal events at the DNA level and understand their downstream consequences.

The current state of software for global profile comparison typically focuses on analyzing and displaying data from a single dimension, for example *CGH Fusion *(infoQuant Ltd, London, UK) for DNA copy number profile analysis and *GeneSpring *(Agilent Technologies, Santa Clara, CA, USA) for gene expression profile analysis. Software for integrative analysis have been restricted to working with datasets derived from limited combination of technology platforms (Table 1) [7-10]. Though different software can analyze data generated from different platforms, the ability to perform meta-analysis using data from multiple microarray platforms is limited to a small number of software packages. Consequently, integrative analysis of cancer genomes typically involves no more than two types of data, most commonly the integration of gene dosage and gene expression data [11-16] and recently expanded to integrating allelic information [17]. Software to perform multi-dimensional analysis are therefore greatly in demand.

**Table 1:** Features required for integrative analysis

*Features required for integrative analysis*	*Nexus CGH*	*CGH Fusion*	*ISA-CGH*	*VAMP*	*CGH Analytics	*MD-SeeGH*	*SIGMA*	*SIGMA*^2^
Built-in segmentation for array CGH	✓	✓	✓	✓	✓	✓		✓
Consensus calling using multiple segmentation algorithms								✓
Array platform-independent combined CGH analysis	✓	✓						✓
Custom microarray data handling	✓	✓	✓	✓	✓	✓		✓
Basic copy number and expression integration			✓	✓	✓			✓
Alignment and analysis of genetic and epigenetic data						✓	✓	✓
Multi-dimensional visualization of genetic, epigenetic and gene expression data								✓
Two group statistical comparison	✓			✓	✓			✓
Two group combinatorial gene dosage and gene expression comparison								✓
Linking to external biological databases	✓	✓	✓	✓	✓	✓	✓	✓
Linking to external gene expression (GEOProfiles)								✓
Context-based visualization of genome features		✓	✓	✓		✓		✓
Conversion of data between different genome assemblies					✓	✓	✓	✓
Free for academic/research use			✓	✓	✓	✓	✓	✓

Here, we present SIGMA^2^, a novel software package which allows users to integrate data from the various 'omics disciplines such as genomics, epigenomics and transcriptomics. Multi-dimensional datasets can be simultaneously compared, analyzed and visualized with respect to individual dimensions, allowing combinatorial integration of the different assays belonging to the different 'omics. The identification of genes altered at multiple levels such as copy number, LOH, DNA methylation and the detection of consequential changes in gene expression can be concertedly performed, establishing SIGMA^2 ^as a tool to facilitate the high throughput systems biology analysis of cancer. SIGMA^2 ^is freely available for academic and research use from our website, .

## Implementation

**Figure 1:**
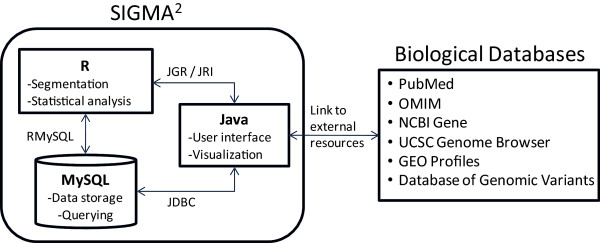
**Main structural components of SIGMA^2^. **Data and genome mapping information is stored in the MySQL database. Segmentation analysis using *DNACopy *and *GLAD *and statistical analysis is performed using R, with results stored in database. Java was used to program the application, specifically for the user interface and the different types of visualization. Base-pair positions and gene annotations are linked to other biological databases to facilitate further interrogation by the user.

SIGMA^2 ^is implemented in Java, and requires version 1.6+ of the runtime compiler. In addition, the statistical package R and database application MySQL are also required. The java interface communicates with MySQL using a JDBC connector and with R using the JRI package by JGR (Figure 1). MySQL is used for data storage and querying while R is used for the segmentation and statistical analysis. All genomic coordinate information was obtained from University of California Santa Cruz (UCSC) genome databases [18].

## Results and discussion

### Look and feel of SIGMA^2^

The novel multi-dimensional 'omics data analysis software SIGMA^2 ^is built on the framework of a facile visualization tool called SIGMA, which can display alignment of genomic data from a built-in static database [7]. The arsenal of functionalities introduced in SIGMA^2 ^is shown in Table 1.

### Description of application scope and functionality

**Figure 2:**
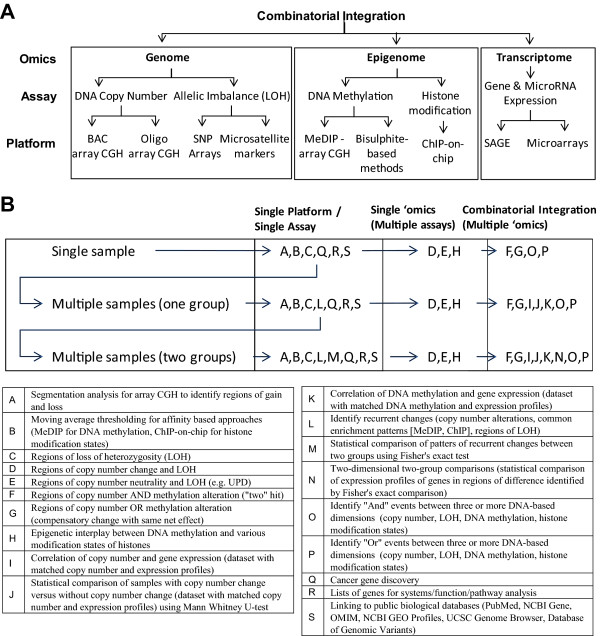
**(A) Data hierarchy describing the relationship between platforms, assays and 'omics disciplines.** (B) Functionality map of SIGMA^2^. List of the various functions and the output from that function that can be performed given the number of samples or sample groups and dimensions. Multiple sample analysis (single group and two group) are microarray platform independent. Functions listed in boxes are in addition to those listed in the box preceding the arrows.

SIGMA^2 ^is built to handle a variety of analysis techniques typically used in the high-throughput study of cancer, allowing the combinatorial integration of multiple 'omics disciplines. The hierarchy, which underlies the program, groups data into genome, epigenome, and transcriptome is shown in Figure 2A and the overall functionality map is given in Figure 2B and listed in Table 2. With each 'omics dimension, data sets may be imported representing any of the major types of biological measurements being assayed, for example, (i) examining both DNA copy number and LOH assays within the genomic bundle, (ii) examining both DNA methylation and histone modification status within the epigenomics bundle, and (iii) examining both gene expression profiles and microRNAs expression assays within the transcriptomic bundle. Each assay may branch into data sources from a multitude of technology platforms.

**Table 2:** Summary of Input, analysis, output for each dimension

*'Omics classification*	*Assay(s) measured*	*Input*	Functionality***	*Output*
Genomics	Copy number	Array CGH	Segmentation Direct thresholding Moving average-based thresholding Z-transformation of moving average Whole genome visualization	Regions of gain and loss Gene lists for further analysis High-resolution karyogram images Frequency histograms
Genomics	LOH	SNPs*	LOH based on consecutive altered markers	Regions of LOH
Genomics	LOH	Microsatellite markers	Same as above	Same as above
Genomics	Copy number, LOH		Identify regions of uniparental disomy (UPD): LOH with no copy number change	
Epigenomics	DNA methylation	MeDIP + array CGH	Direct thresholding Moving average-based thresholding Z-transformation of moving average	Regions of enrichment and lack of methylation Gene lists for further analysis
Epigenomics	DNA methylation	Bilsulphite-based	Visualization against genome position Thresholding of proportion of methylated CpG's	
Epigenomics	Histone modification states	ChIP-on-chip	Direct thresholding Moving average-based thresholding Z-transformation of moving average	Regions of enrichment and lack of enrichment Gene lists for further analysis
Epigenomics	DNA methylation, Histone modification states		Epigenetic interplay	Regions of mutually exclusive change between chromatin state and DNA methylation
Transcriptomics	Gene expression**	Microarrays	Heatmap visualization, clustering Histograms Statistical comparisons	Expression of genes of interested based on DNA analysis
Transcriptomics	Gene expression**	SAGE	Heatmap visualization, clustering Histograms Statistical comparisons	Expression of genes of interested based on DNA analysis
Genomics, Transcriptomics	Copy number, Gene expression		Correlation analysis of copy number and expression Statistical comparison of expression in regions of copy number difference (two group analysis)	Genes whose expression is strongly regulatd by copy number p-values for associations p-values for group comparison
Genomics, Epigenomics	Copy number, DNA methylation		Identify regions of concerted change in BOTH copy number and methylation ("two-hit") Identify regions with change in copy number OR DNA methylation	
Genomics, Epigenomics	LOH, DNA methylation		Identify allele-specific methylation events	Regions of allele specific aberrant methylation
Genomics, Epigenomics, Transcriptomics	Copy number, LOH, DNA methylation, Histone modification Gene Expression		Identify co-ordinate genetic, epigenetic and gene expression changes	Genes altered at multiple levels

* Affymetrix and Illumina data must be pre-processed prior to import; ** functionality invoked in the context of genetic and epigenetic data analyses; ***aligned to genome features (Database of genomic variants, CpG Islands, microRNAs etc.)

### Approach to integration between array platforms and assays

**Figure 3:**
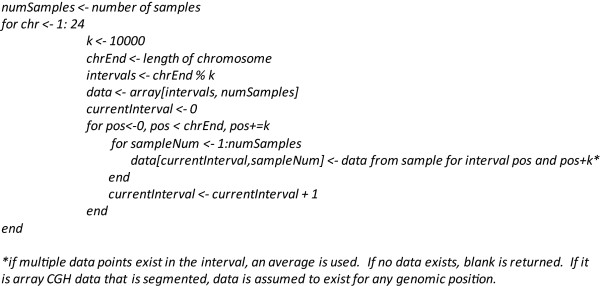
**Algorithm for integrating between different array platforms.** Data for every platform is matched to genomic position. Subsequently, an interval-based approach is used to systematically query data for each interval. In this figure, the interval, k, is 10 kb in size. By converting everything to genomic position, samples sets of the same disease type but on different array platforms can be aggregated affording the user with additional statistical power.

SIGMA^2 ^treats all data in the context of genome position based on the relevant human genome build using the UCSC genome assemblies. An interval-based approach is used to sample across different array platforms and assays and data from each interval are merged together. Briefly, this is done by querying data at fixed genomic intervals for each platform and subsequently taking an average of the measurements within each interval. The algorithm is listed in Figure 3.

### Format requirements of input data

Standard tab-delimited text files are used for the input of data for all of the assay types. For genomic data, specifically array CGH, normalization is recommended using external algorithms such as *CGH-Norm *and *MANOR *[19,20]. Segmentation analysis can be performed within SIGMA^2^, but results from external analysis can be imported and used in the consensus calling feature. The algorithms which can be called within SIGMA^2 ^currently include *DNACopy *and *GLAD *[21,22]. Multiple sample batch importing is available to facilitate efficient loading of datasets. To utilize this, the user must create an information file which describes each sample in the dataset. Formatting requirements of the information file are specified in the manual. Alternatively, for Affymetrix SNP array analysis, data should also be pre-processed and normalized using the appropriate software, such as *CNAG *before importing into SIGMA^2 ^[23]. Genotyping calls should be made prior to importing, using the "AA", "AB" and "BB" convention. If the genotype call does not exist, "NC" must be specified. For epigenomic data, data from affinity based-approaches (MeDIP [6] and ChIP [24]) should contain a value representing the level of enrichment and the genomic coordinates for each spot. Similarly, for bisulphite-based approaches [25], a percent of converted CpGs should be provided along with the genomic coordinates for each spot. Finally, for transcriptome data, gene expression data from Affymetrix experiments can be directly imported and processed as CEL files and are normalized using the MAS 5.0 algorithm implemented in the "affy" package of R. For any assay type, custom data can be imported whereby the user provides a map of the platform based on the given genome build, and the unique identifier for the map must be used for the data generated from those experiments.

### Description of user interface

**Figure 4:**
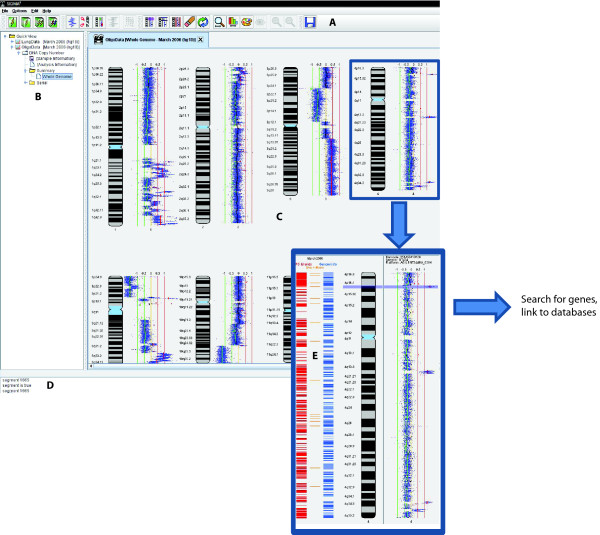
**Description of the SIGMA^2 ^user interface using a single sample visualization as an example.** (A) Customizable toolbar with shortcut buttons, (B) Project/Analysis tree to track work within and between sessions, (C) Main display area using tab-based navigation, (D) Information console and (E) Genome features tracks. Here, a copy number change is displayed in the context of CpG islands (red), microRNAs (orange) and regions annotated in the database of genomic variants (blue).

The main user interface in SIGMA^2 ^utilizes a tabbed window-pane which allows the user to open multiple visualizations simultaneously (Figure 4). The left part of the window manages the analyses and projects which belong to the current user and button shortcuts for the main functionality are spread along the top of the window. Using an example of an array CGH profile from the Agilent 244K platform, we demonstrate the step-wise interrogation of a region of interest [26]. Briefly, using the highlighting toolbar button, the user can select a region of interest and subsequently, by clicking the right mouse button, the user can search for annotated genes within the specified genomic coordinates.

### Analysis of data from a single assay type

**Figure 5:**
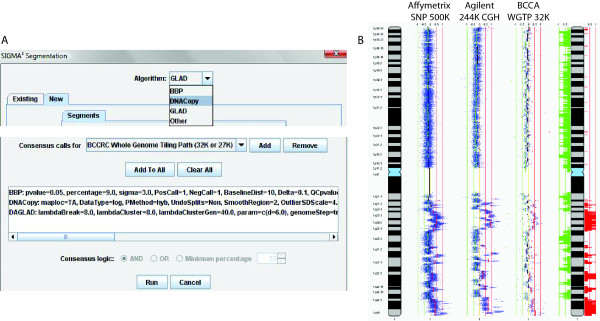
**(A) Consensus calling using multiple algorithms. Multiple algorithms (and different parameters) can be selected to analyze a given array CGH sample and this can be defined for each array platform independently as each platform may have exhibit different noise and ratio response characteristics.** (B) Heterogeneous array analysis using data from multiple array CGH platforms. Sample from the Agilent 244K, Affymetrix SNP 500K and whole genome BAC array were segmented to define areas of gain and loss. Subsequently, the results were aggregated into a frequency histogram plot showing the common areas of gain and loss across the three samples.

The first, and most basic, level of analysis is from a single assay type. For array CGH, multiple options for segmentation algorithms are available within the program and results from externally run segmentation can be imported as well. However, each segmentation algorithm has its advantages and disadvantages depending on the type of data used and the quality of data at hand. A unique feature of SIGMA^2 ^is the ability to take a consensus of multiple algorithms using "And" or "Or" logic between algorithms. Moreover, a level of consensus can be specified (Figure 5A). For example, if an experiment is analyzed using five approaches, the user can select areas of gain and loss which were detected by one algorithm, at least three algorithms, all five algorithms, etc. For LOH, basic analysis using the number of consecutive markers that exhibit LOH is used to determine its status. Affinity-based approaches for DNA methylation and histone modification states or bead-based percentage of CpG island methylation is analyzed by either direct thresholding or z-transform thresholding. For any of the different assay types, when examining across a number of samples, a frequency of alteration can be calculated and plotted.

For data from different array platforms, but assaying the same biological measurement, the algorithm for integration is used to derive common data. This feature is most applicable to DNA copy number data due to the number of array CGH platforms. This allows for better utilization of publicly available data and thus, increasing sample size for statistical analysis. Similar to the multiple sample analysis of data on the sample platform, a frequency of altered states can be generated and plotted. Figure 5A shows the concerted analysis of a sample profiled on the Affymetrix 500K SNP array, Agilent 244K CGH array and the whole genome tiling path BAC array (Figure 5B).

### Analysis of data from multiple assays in a given 'omics dimension

**Figure 6:**
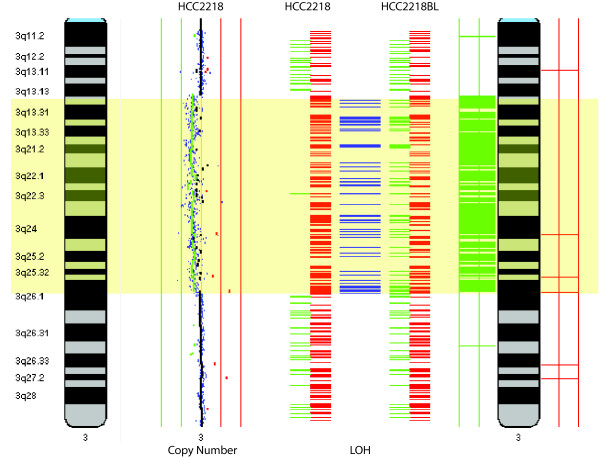
**Parallel visualization and analysis of the copy number and genotype profiles of the breast cancer cell line HCC2218.** Genotype profile of the matching normal blood lymphoblast line (HCC2218BL) is also provided to define regions of LOH. DNA copy number profile was generated on the BCCA whole genome tiling path BAC array and genotype profiles are from the Affymetrix SNP 10K array [28]. This region of chromosome arm 3q has a defined segmental copy number loss and the boundary of the change is evident from the LOH profile. In the genotype profile, the horizontal blue lines indicate a SNP transition from heterozygous in normal to homozygous in the tumor, indicating LOH.

Within a given 'omics dimension, multiple assay types can be analyzed in combination. For example, it is useful to investigate copy number and LOH and the interplay between DNA methylation and different states of histone modification. Typically, in regions of copy number loss, LOH is also observed. However, LOH can also occur in regions which are copy number neutral, indicating a change in allelic status which is not interpretable by one dimension alone. Here, we show a sample for which copy number and LOH information exists, a region of copy number loss associated with LOH (Figure 6). In terms of epigenetics, DNA methylation and states of histone methylation and acetylation have been known to be biologically relevant. With high throughput technologies available to assay these dimensions, this type of analysis will become more prevalent.

### Combinatorial analysis of multiple 'omics dimensions – gene dosage and gene expression

The most common analysis of multiple 'omics dimensions is the influence of the genome on the transcriptome. A number of software packages have started to incorporate approaches to examining gene dosage and gene expression [8,9,27]. In SIGMA^2^, there are multiple functionalities which allow the user to link DNA copy number to gene expression. For a single group of samples, with matching DNA copy number and gene expression profiles, the user can determine associations through two main options: a) using a correlation-based approach, correlating the log ratios with the normalized gene expression intensities and b) using a statistical-based approach comparing the expression in samples with copy number changes against those without copy number change utilizing the Mann Whitney U test, analogous to approaches taken in previous studies [27]. Spearman, Kendall or Pearson correlation coefficients can be calculated for option a). Similarly, this functionality is also available for correlating epigenetic profiles and gene expression.

**Figure 7:**
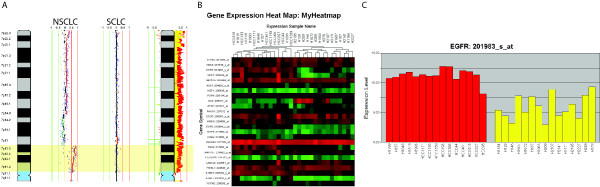
**A two-group two dimensional comparison of 37 NSCLC and 16 SCLC cancer cell lines.** First, segmentation analysis is performed to delineate gains and losses in each sample. Next, a statistical comparison of the distribution of gains and losses between the two groups is done using the Fisher's exact test. (A) Using the interactive search, one of the regions of difference identified is on chromosome 7, with a NSCLC and SCLC sample aligned next to each other. The NSCLC has a clear segmental gain of that region, with the SCLC not having the gain. The right-most graph is a frequency plot summary of two sample sets (NSCLC and SCLC). NSCLC is color-coded in red while SCLC in green, and the overlap appears in yellow. The frequency of chromosome arm 7p gain is higher in the red group. (B) A heatmap is shown representing 15 NSCLC and 15 SCLC gene expression profiles, of the specific genes in the region highlighted in yellow. (C) When examining gene expression data of EGFR specifically, a gene in this region, we can see that the expression is drastically higher in NSCLC vs. SCLC, as predicted by the higher frequency of gain in NSCLC vs. SCLC of that region. Gene expression data are represented as log2 of the normalized intensities.

In addition to single group analysis, two-dimensional genome/transcriptome analysis can be applied to two-group comparison analysis. For example, if patterns of copy number alterations are compared between two groups and a particular region is more frequently gained in one group than another, the expression data can subsequently compared between the groups of sample to determine if there is an association between gene dosage and gene expression. That is, we would expect the group with more frequent copy number gain to have higher expression than the other group. Notably, this functionality does not require both copy number and expression data to exist for the same sample, but allows the user to select an independent dataset for expression data comparisons (Figure 7).

### Group comparison analysis – single 'omics dimension

Finally, for two groups of samples, the user can compare the distribution of changes between two groups to determine if the patterns are statistically different using a Fisher's Exact test. For DNA copy number, it is the distribution of gain and losses; for DNA methylation or histone modification states, the proportion of samples that meet the threshold of enrichment for each group (low or high); and for LOH, proportion of samples with LOH for a region for each group.

### Group comparison analysis – integrating multiple 'omics dimensions

This type of analysis can be performed with a single sample or multiple samples, thus allowing combinatorial ("and") analysis for large datasets. In addition, the user can also identify "or" events, where a change in any of the dimensions can be flagged. This is more important in multi-sample datasets as one dimension may not capture complex alterations of a particular region.

### Multi-dimensional analysis of a breast cancer genome

**Figure 8:**
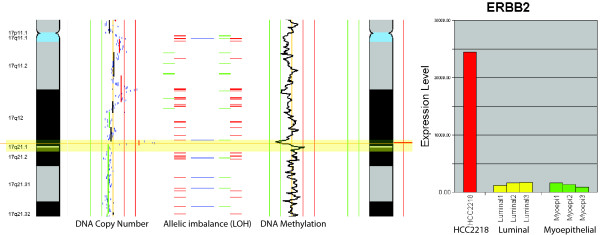
**Multi-dimensional perspective of chromosome 17 of the HCC2218 breast cancer cell line.** Copy number, LOH, and DNA methylation, and profiling identifies an amplification of ERBB2 coinciding with allelic imbalance and loss of methylation. When examining the gene expression, the expression of HCC2218 is significantly higher than a panel of normal luminal and myoepithelial cell lines [29].

Using the breast cancer cell line HCC2218, we show the integration of genomic, epigenomic, and transcriptomic data. Interestingly, when we examine the *ERBB2 *gene on chromosome 17, we show concurrent amplification, LOH, loss of methylation and drastic increase in gene expression (Figure 8). *ERBB2 *has shown to be an important gene in breast cancer development and therapeutic intervention. This demonstrates the value in integrating multiple dimensions to understand complex alteration patterns in disease samples where multiple causes can lead to a single effect.

### Exporting data and results

High resolution images can be exported for all types of visualizations in SIGMA^2^. Histogram plots of gene expression, heatmaps with clustering of gene expression, karyogram plots and frequency histogram plots are the main types of visualization available. Frequency histogram data which is used to generate the plots can also be exported. Integrated plots with data plotted serially or overlaid are also available for analysis involving multiple genomic and epigenomic dimensions. Genes which are obtained from the conjunctive (And) and disjunctive (Or) multi-dimensional analysis can be exported with their status. Results of statistical analysis such as Fisher's exact comparisons and U-test comparisons of gene expression can be exported against annotate gene lists based on user-specified human genome builds. Currently, April 2003 (hg15), May 2004 (hg17) and March 2006 (hg18) are the available genome builds [18]. As new builds are released, support for those builds will be available. Finally, data from multi-platform integration can be exported based on based pair position for additional external analysis if necessary.

## Conclusion

With the increase in high-throughput data covering multiple dimensions of the genome, epigenome and transcriptome, the approaches and tools to analyze this data must advance accordingly to handle, analyze and interpret this data in an integrated manner. SIGMA^2 ^meets these requirements and provides the framework for the incorporation of data from future approaches and technologies. Specifically, with the movement from array to sequence-based technologies, the ability to assimilate sequence data with the various 'omics data sets will become a future requirement of software packages.

## Availability and requirements

Project name: SIGMA^2^

Operating system(s): Java SE V.1.6+, R Project V.2.5+, Windows XP or Vista

License: Free for academic and research use; commercial users please contact

## Authors' contributions

RC designed and developed the software and wrote the manuscript. BPC contributed to the design and development of the software. CW and MB contributed significantly to software development. IMW and EAV contributed to beta testing and ideas for refinement of software. CM and RTN contributed concepts for implementation of data integration and statistical analyses. WLL is the principle investigator of this study. All authors contributed to the critical reading and editing of the manuscript.
